# Supplementary material for: During natural viewing, neural processing of visual targets continues throughout saccades
Source: J Vis. 2021 Sep 7;21(10):7. doi: 10.1167/jov.21.10.7 (PMC8431980; doi:10.1167/jov.21.10.7)
Supplement: Supplement 1 [file jovi-21-10-7_s001.pdf]

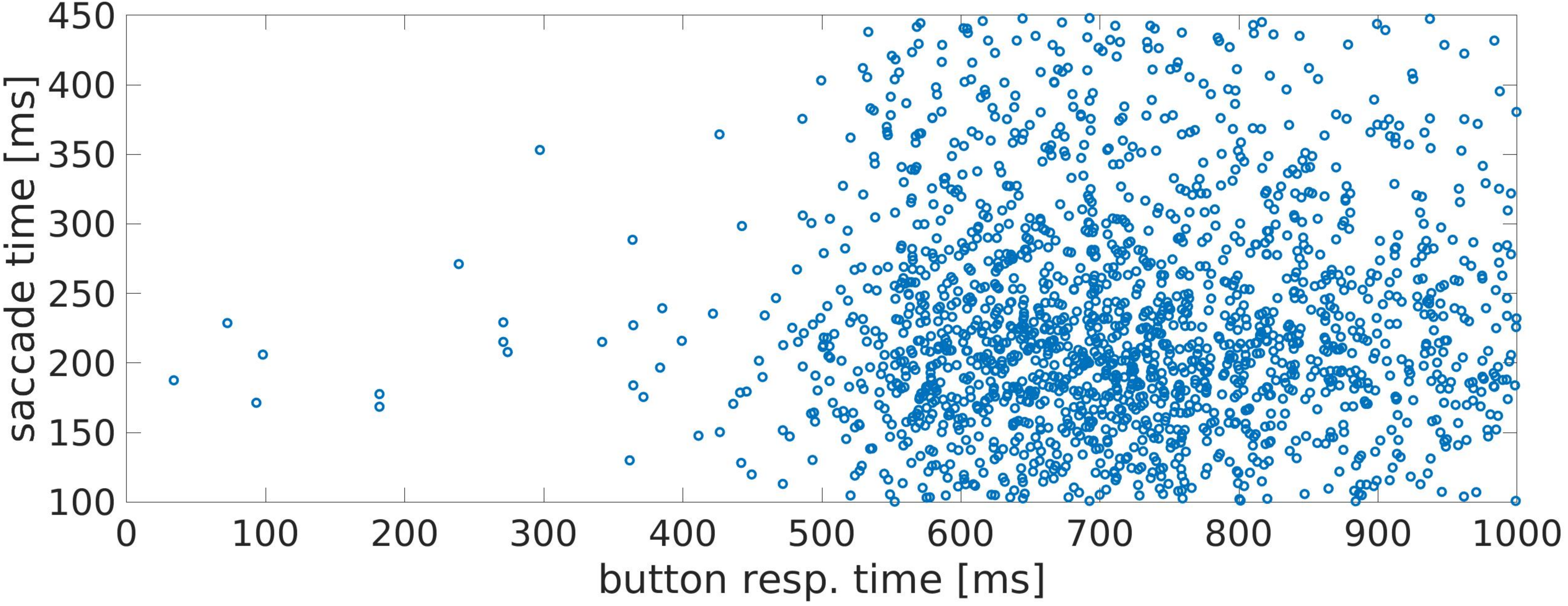

Fig. S1: Button press supplement: A: Button press TRF **B:** Scatter plot of all saccades to target times plotted against button response times. There was no relationship between when subjects looked towards a target and how fast they responded to it (Spearman correlation: r=0.012, p=0.59, N=1749).
