# Supplementary material for: During natural viewing, neural processing of visual targets continues throughout saccades
Source: J Vis. 2021 Sep 7;21(10):7. doi: 10.1167/jov.21.10.7 (PMC8431980; doi:10.1167/jov.21.10.7)
Supplement: Supplement 2 [file jovi-21-10-7_s002.pdf]

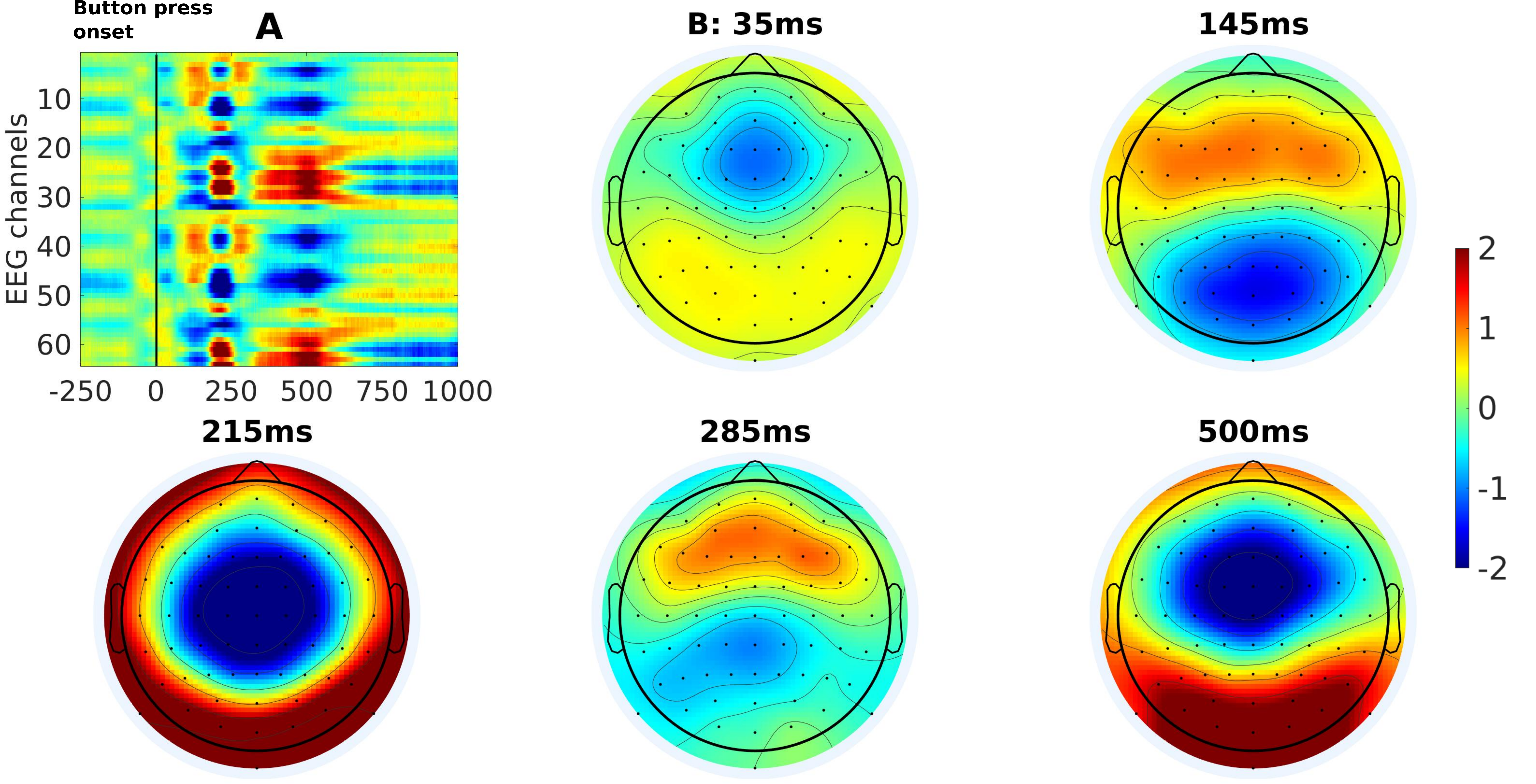

Fig. S2: Button press response TRF A: Response across all 64 channels locked to button press at 0ms. **B:** Representative topographic plots of post-button press peaks at 35, 145, 215, 285 and 500ms.
