# Supplementary material for: During natural viewing, neural processing of visual targets continues throughout saccades
Source: J Vis. 2021 Sep 7;21(10):7. doi: 10.1167/jov.21.10.7 (PMC8431980; doi:10.1167/jov.21.10.7)
Supplement: Supplement 3 [file jovi-21-10-7_s003.pdf]

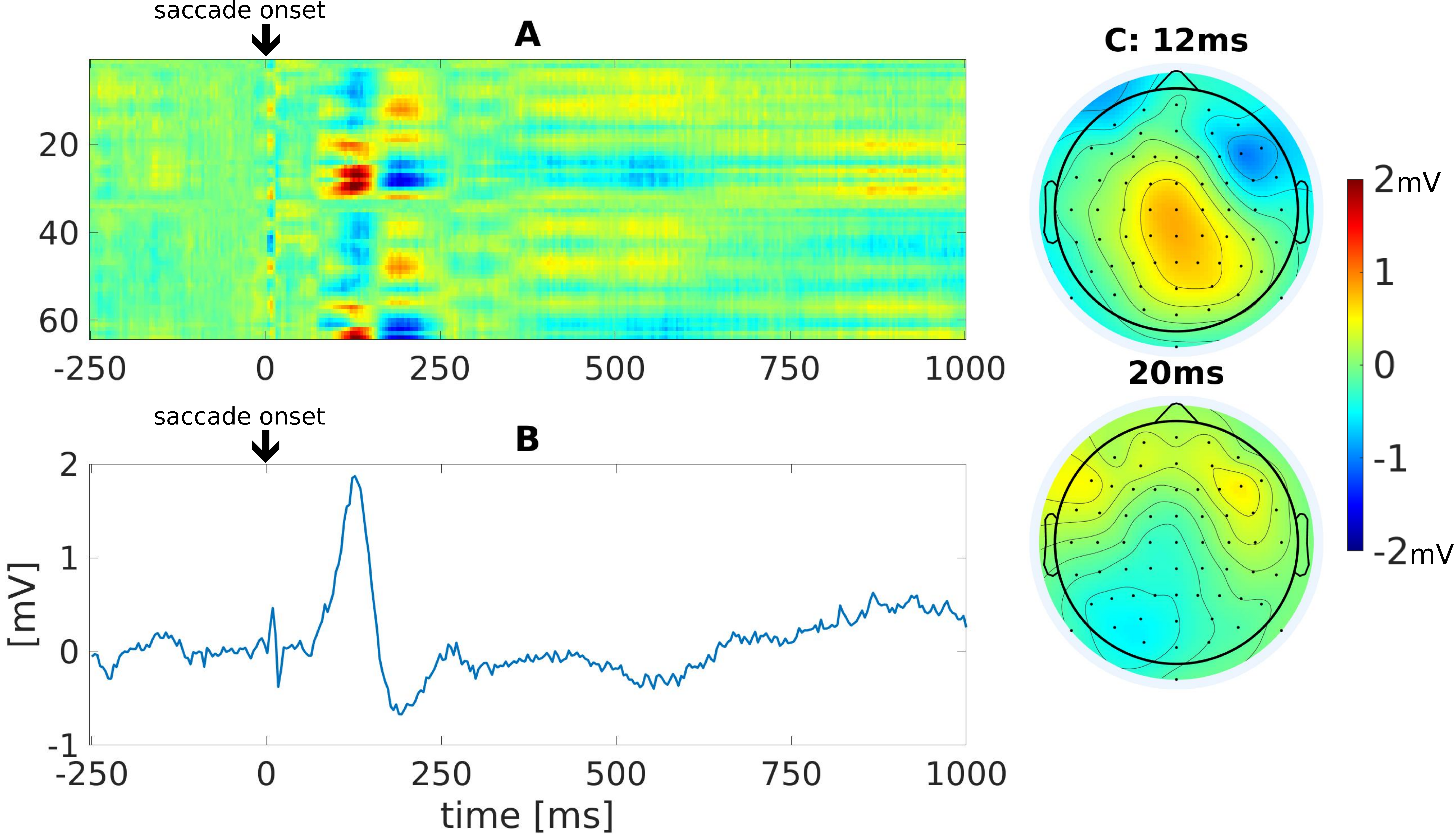

Fig. S3: Saccade-locked TRF of all targets showing the stereotypical lambda complex A: TRF locked to target presentations (demarcated by 0 ms). **B:** Average trace of electrodes CPz, POz, and Oz. **C:** Respective topographic snapshot of peaks at 12ms and 20ms. Peak at 12ms is indicative of the spike potential of the saccadic lambda complex.
