# Supplementary material for: During natural viewing, neural processing of visual targets continues throughout saccades
Source: J Vis. 2021 Sep 7;21(10):7. doi: 10.1167/jov.21.10.7 (PMC8431980; doi:10.1167/jov.21.10.7)
Supplement: Supplement 4 [file jovi-21-10-7_s004.pdf]

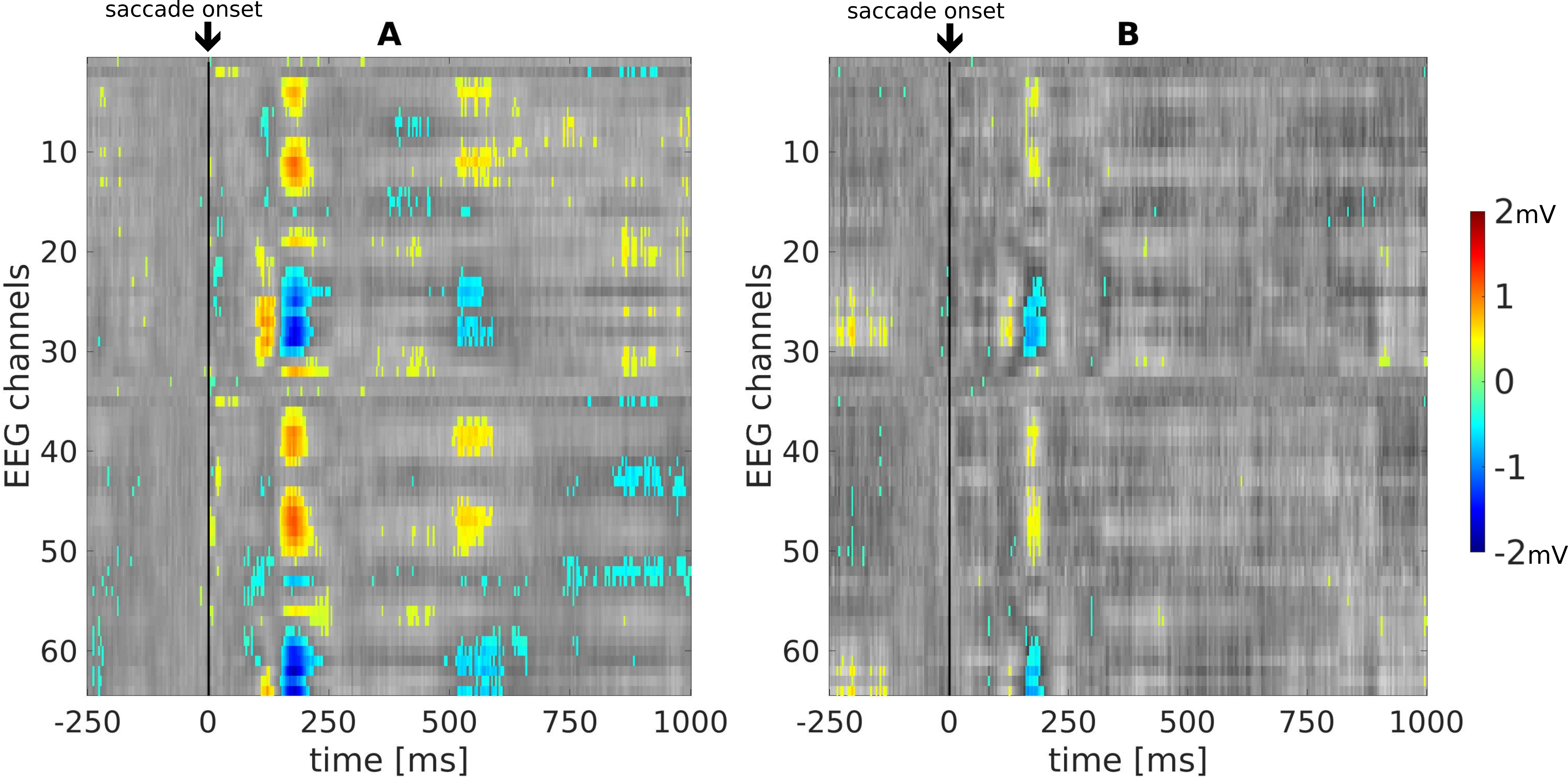

Fig. S4: Target-elicited saccades and spontaneous saccades contrast A: Saccade-locked TRF shows a replicable difference for both “easy” and (target: N=1749, spontaneious: N=12437) **B:** “hard” game conditions (target: N=1774, spontaneous: N=13937).
