# Supplementary material for: During natural viewing, neural processing of visual targets continues throughout saccades
Source: J Vis. 2021 Sep 7;21(10):7. doi: 10.1167/jov.21.10.7 (PMC8431980; doi:10.1167/jov.21.10.7)
Supplement: Supplement 5 [file jovi-21-10-7_s005.pdf]

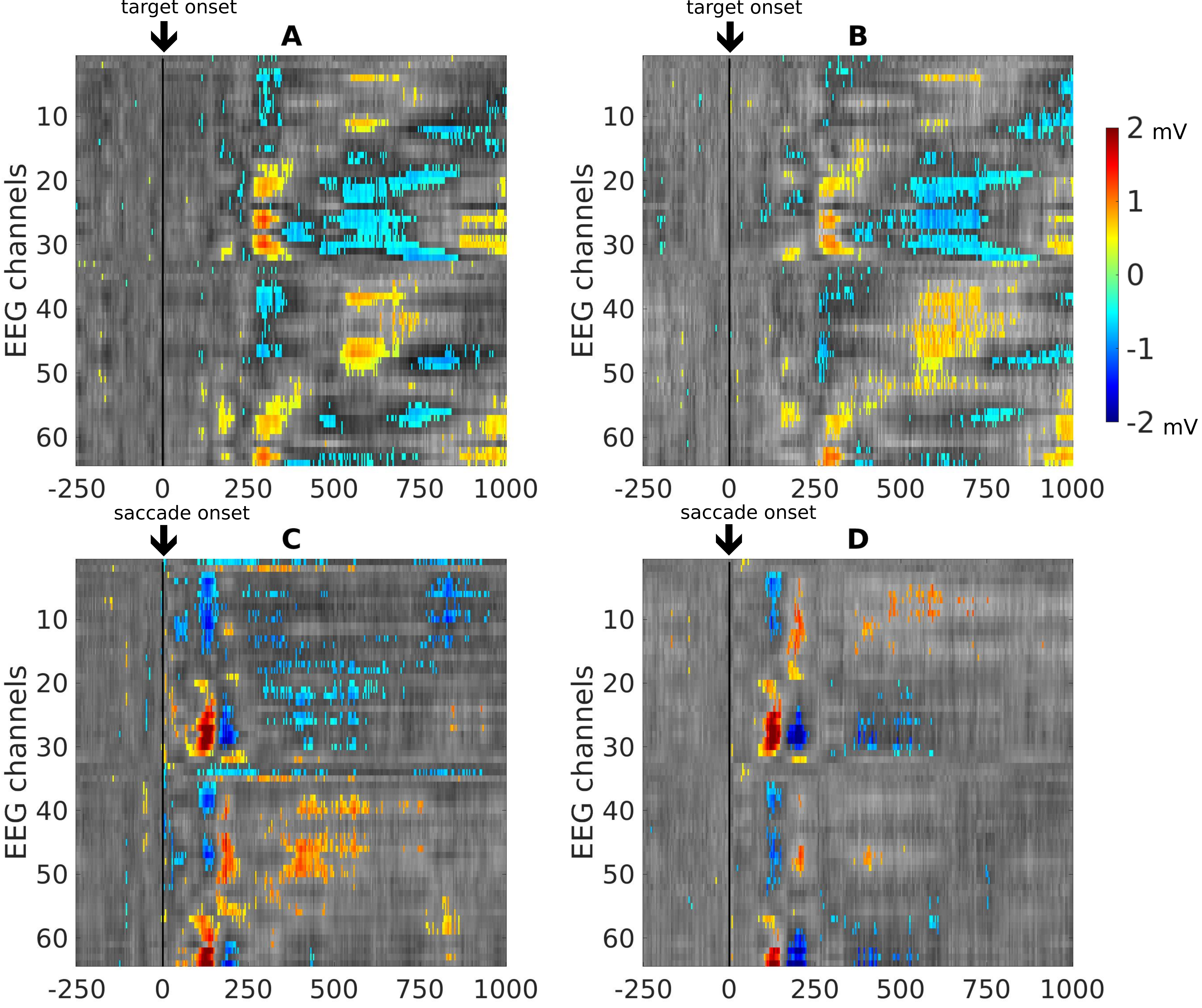

Fig. S5: Target visibility supplement: A: Target-locked TRF differences between clear and for the “easy” game condition replicates with the (clear: N=3159, fog: N=876) **B:** target-locked “hard” game condition (clear: N=2784, fog: N=619). **C:** Similarly the saccade-locked TRF shows a replicable difference for both “easy” and (clear: N=1382, fog: N=367) **D:** “hard” game conditions (clear: N=1461, fog: N=313).
