# Supplementary material for: During natural viewing, neural processing of visual targets continues throughout saccades
Source: J Vis. 2021 Sep 7;21(10):7. doi: 10.1167/jov.21.10.7 (PMC8431980; doi:10.1167/jov.21.10.7)
Supplement: Supplement 6 [file jovi-21-10-7_s006.pdf]

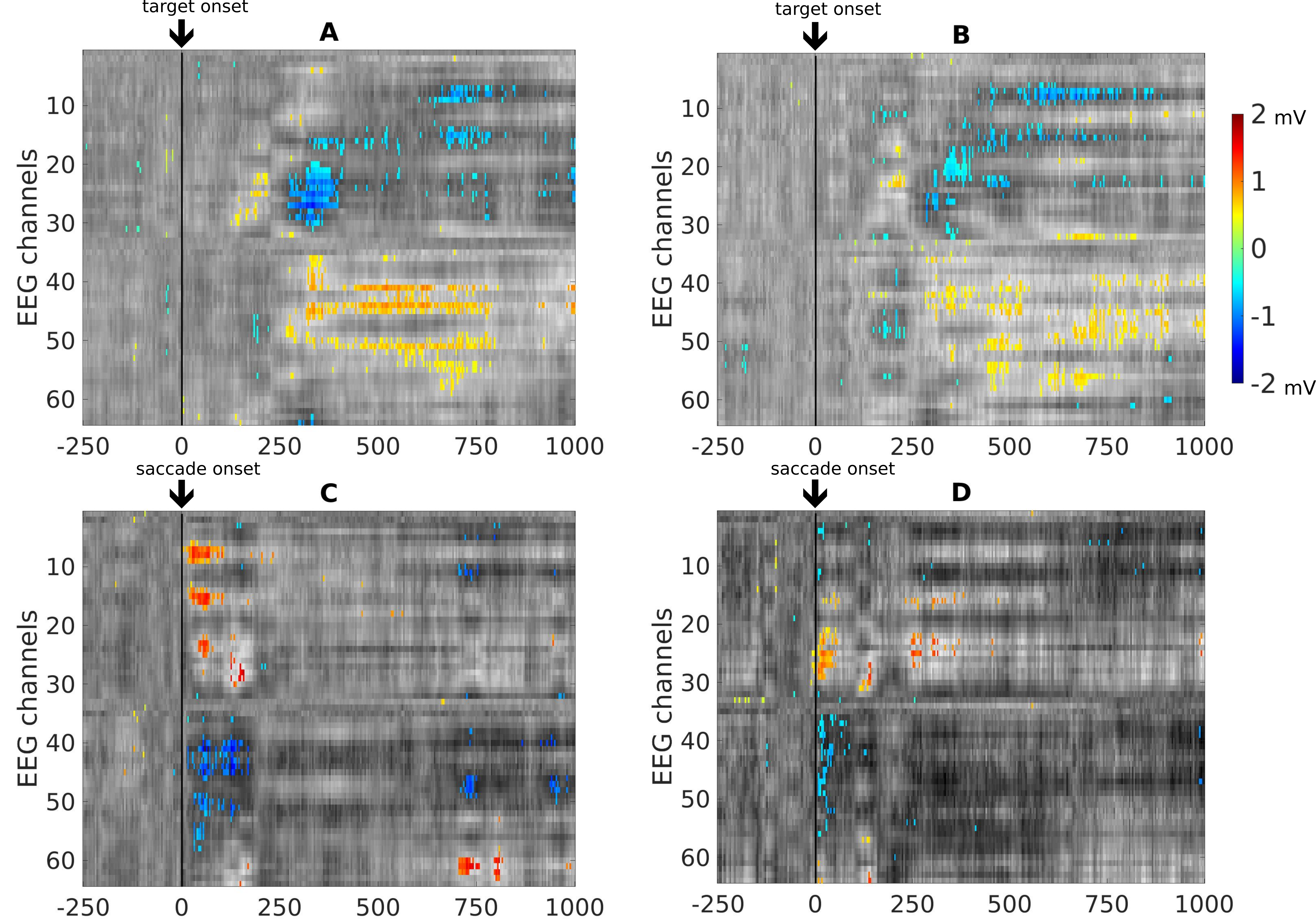

Fig. S6: Target location supplement: A: Target-locked TRF differences for shows a strong late contrast between peripheral and central for the “easy” game condition (peripheral: N=1546, central: N=1613) which replicates with the **B:** target-locked “hard” game condition (peripheral: N=1310, central: N=1474). **C:** Saccade-locked TRF shows a replicable difference for both “easy” (peripheral: N=830, central: N=552) and **D:** “hard” (peripheral: N=838, central: N=623) game condition.
