# Supplementary material for: During natural viewing, neural processing of visual targets continues throughout saccades
Source: J Vis. 2021 Sep 7;21(10):7. doi: 10.1167/jov.21.10.7 (PMC8431980; doi:10.1167/jov.21.10.7)
Supplement: Supplement 7 [file jovi-21-10-7_s007.pdf]

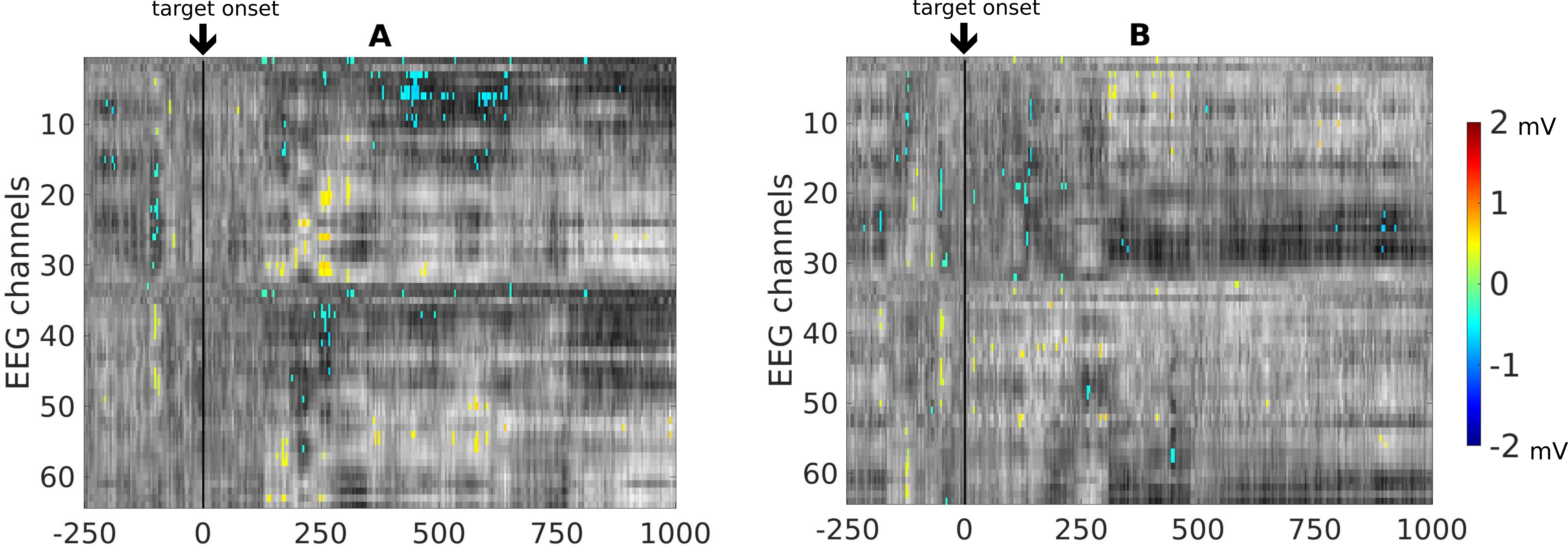

Fig. S7: Threat contrast supplement: A: "easy" game condition (threat: N=1571; no-threat: N=1588) showing significance between 175-250ms and 430-645 but this does not replicate for the **B:** "hard" game conditions (threat: N=1328; no-threat: N=1456). Saccade-locked results showed no significance.
